# Dissolved Concentrations, Sources, and Risk Evaluation of Selected Metals in Surface Water from Mangla Lake, Pakistan

**DOI:** 10.1155/2014/948396

**Published:** 2014-03-09

**Authors:** Muhammad Saleem, Javed Iqbal, Munir H. Shah

**Affiliations:** Department of Chemistry, Quaid-i-Azam University, Islamabad 45320, Pakistan

## Abstract

The present study is carried out for the assessment of water quality parameters and selected metals levels in surface water from Mangla Lake, Pakistan. The metal levels (Ca, Cd, Co, Cr, Cu, Fe, K, Li, Mg, Mn, Na, Ni, Pb, Sr, and Zn) were determined by flame atomic absorption spectrophotometry. Average levels of Cd, Co, Cr, Ni, and Pb were higher than the allowable concentrations set by national and international agencies. Principal component analysis indicated significant anthropogenic contributions of Cd, Co, Cr, Ni, and Pb in the water reservoir. Noncarcinogenic risk assessment was then evaluated using Hazard Quotient (HQ_ing/derm_) and Hazard Index (HI_ing/derm_) following USEPA methodology. For adults and children, Cd, Co, Cr, and Pb (HQ_ing_ > 1) emerged as the most important pollutants leading to noncarcinogenic concerns via ingestion route, whereas there was no risk via dermal contact of surface water. This study helps in establishing pollutant loading reduction goal and the total maximum daily loads, and consequently contributes to preserve public health and develop water conservation strategy.

## 1. Introduction

Lakes have important multiusage components, such as sources of drinking water, irrigation, shipping, fishery, landscape entertainment, and energy production [1]; the Mangla Lake located in Mirpur district, Pakistan, is no exception. However, the quality of water is a very sensitive issue and numerous anthropogenic (e.g., urban, industrial, and agricultural activities) as well as natural processes (e.g., changes in precipitation inputs, erosion, and weathering of crustal materials) degrade surface water and impair its use for drinking, industrial, agricultural, and recreation purposes [2–5]. Water pollution with toxic metals due to anthropogenic processes is of great concern worldwide [6–11]. Metal pollutants in aqueous system are often recycled via physiochemical and biological processes, which continue to pose a risk of adverse effects on human health, water, soil quality, and the crops [12–14]. Therefore, researchers worldwide focus their attention on quantitative investigation of the trace metals in aquatic ecosystems [15–17].

The major objective of the present study was to assess surface water quality from Mangla Lake and to compare the measured levels of the studied parameters with national and international water quality guideline values. Hence, the study was based on the measurement of existing levels of water quality parameters, including pH, dissolved oxygen (DO), total alkalinity (TA), electrical conductivity (EC), total dissolved solids (TDS), and chloride (Cl^−^), and selected metals (Ca, Cd, Co, Cr, Cu, Fe, K, Li, Mg, Mn, Na, Ni, Pb, Sr, and Zn) in surface water in summer and winter from Mangla Lake. Multivariate principal component analysis (PCA) was employed in order to find out the plausible contributing sources of selected metals in the water samples. Human health risk assessment was carried out to evaluate adverse health risks associated with exposure to these metals via oral ingestion and absorption through the skin for children and adults.

## 2. Materials and Methods

### 2.1. Study Area

**Figure 1:**
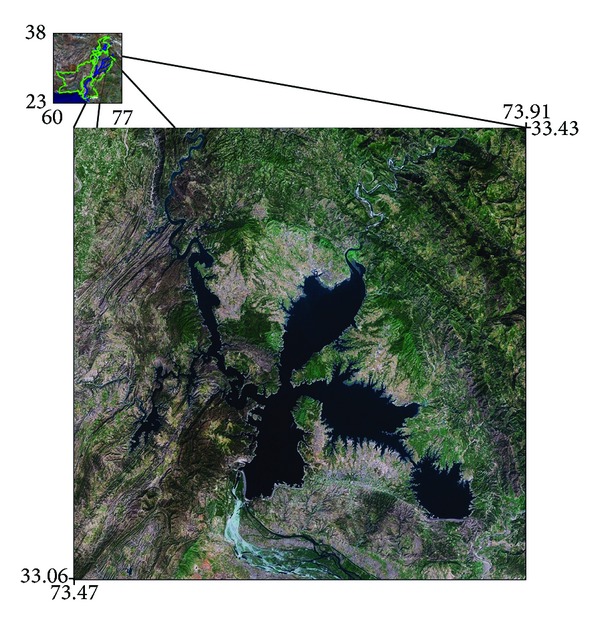
Location map of the study area.

Mangla Lake (Mirpur, Azad Kashmir, Pakistan) is the 12th largest lake in the world. The lake is approximately 100 km south-east of the capital city, Islamabad, Pakistan. The lake (longitude: 73.65 (73° 39′ 0 E) and latitude: 33.15 (33° 8′ 60 N)) was constructed in 1967, across the Jhelum River, in Mirpur District of Azad Kashmir, Pakistan (Figure 1). Other major rivers that contribute to the water storage are Neelum, Kunhar, and Poonch. It has six reservoir pockets: Jhelum, Kanshi, Poonch, Main, Khud, and Jari. The main structures of the lake include 4 embankment lakes, 2 spillways, 5 power-cum-irrigation tunnels, a 1000 MW power station, and upper Jhelum canal. The main lake is 3140 m long and 138 m high (above core trench) with a reservoir of 253 km² [18]. Since its construction, the water storage capacity of Mangla Lake has been reduced from 7,254.74 to 5,764.31 million cubic meters due to the sedimentation [18, 19].

The lake has already contributed significantly towards improvement of the environment in terms of agriculture growth, job opportunities, and improved standard of living. Many thousands of acres of land are irrigated using this water. Availability of additional water and hydropower will further enhance these positive impacts. The Mangla Lake was designed primarily to increase the amount of water that could be used for irrigation from the flow of the Jhelum River and its tributaries. Its secondary function was to generate electrical power from the irrigation releases at the artificial head of the reservoir [18, 20]. Recently, water from the reservoir is being used for water supply to the surrounding areas.

### 2.2. Sample Collection, Processing, and Analysis

Triplicate surface water samples (*n* = 150) in each season were collected in polyethylene bottles (1.5 L capacity) through direct method following standard methodology [21]. Every water sample was collected by combining three fractions of equal volume, each of which was collected from an area of 10–20 m^2^. The water samples were filtered (0.45 *μ*m, pore size) to remove the suspensions. The initial portion of the filtration was discarded to clean the filter surface, and the following ones intended for metal analysis were acidified to pH < 2 using nitric acid and then stored in refrigerator in precleaned polyethylene bottles until analysis.

Standard methods of analyses were adopted for the measurement of water quality parameters [21–23]. The pH, EC, TDS, and DO of each water sample were measured at the sampling points by digital pH, EC, and DO meters, respectively [23]. The Cl^−^ and total alkalinity (TA) contents were determined by the standard method [22]. The water samples were analyzed for Ca, Cd, Co, Cr, Cu, Fe, K, Li, Mg, Mn, Na, Ni, Pb, Sr, and Zn under optimum analytical conditions (Table 1) using a Shimadzu Atomic Absorption Spectrophotometer (Model AA-670, Japan) equipped with automatic background compensation. Calibration line method using five standards was employed for the quantification of selected metals [23, 24]. Standard reference material (SRM-1643d) was also used to ensure the reliability of the metal data (Table 2).

All the reagents used were of analytical grade (certified purity > 99.99%) procured from E-Merck, Darmstadt, Germany, or BDH, UK. Doubly distilled water was used for the preparation of standards and the dilution of samples whenever required [23, 24]. The metal standards were prepared from stock solution of 1000 mg/L by successive dilutions. For the removal of inorganic/organic impurities from glassware, they were first washed with tap water, then washed with 5% (w/v) detergent solution, afterwards soaked in 5% (v/v) nitric acid for overnight, and finally rinsed with plentiful distilled water. If some adhering organic matter was suspected, a final rinse with acetone was given. The glassware was then dried in an electric oven maintained at 80°C for about six hours prior to use. All the measurements were made in triplicate.

### 2.3. Statistical Analysis

Statistical methods were applied to process the analytical data in terms of its distribution and correlation among the studied parameters. STATISTICA software was used for the computation of the data [25]. Basic statistical parameters such as minimum, maximum, mean, median, standard deviation, and skewness were calculated, while multivariate statistic in terms of principal component analysis (PCA) was also carried out using varimax-normalized rotation on the dataset [23, 26]. PCA is mainly used for data reduction and it aims at finding a few components that explain the major variation within the data.

## 3. Results and Discussion

### 3.1. Distribution of Water Quality Parameters in Surface Water

The suitability of water samples mainly depends upon the mineral constituents present in the water. The major quality criteria parameters are pH, DO, TA, EC, TDS, chloride, some metals, and so forth, [27]. Descriptive statistics related to water quality parameters in summer and winter along with water quality guidelines are described in Table 3. The pH levels of the water varied as 7.6–8.2 and 7.4–8.3 with mean values of 8.0 and 7.8 in summer and winter, successively. It indicated that the water was generally alkaline in nature and the dissolved carbonates were predominantly in the form of HCO_3_ [28]. Moreover, pH levels were within the permissible limits set by international authorities [27, 29, 30]. Higher levels of pH can decrease the solubility of Cd, Cu, and Pb, while lower pH levels can dissolve metal complexes, releasing free metal ions into the water column [31]. The measured pH values were higher than those reported in Pandoh Lake, India [32], and Bozkowo, Domimickie, and Wiekie Lakes in W. Poland [33] (Table 4). Mean values of total alkalinity were lower than the respective water criterion [27] (Table 3). Average measured temperature values were 38 and 11°C in summer and winter, respectively. Mean DO levels were found to be 4.3 and 4.4 in summer and winter, respectively. The measured levels were far lower than those reported in Pandoh Lake, India [32], and Lake Beysehir, Turkey [34]. Variability of DO can be related to flow regime, seasonal effects, and anthropogenic impacts [35]. A warm water aquatic ecosystem should have dissolved oxygen concentration of at least 5 mg/L in order to support the diversified biota [22]. Untreated discharge of municipal effluents, solid wastes from villages, nearby towns and cities, and wastes released from poultry farms in catchment areas may be possible reasons of lower DO.

The total dissolved solids (TDS) and electrical conductivity (EC) are important parameters as they can affect taste of water, in addition to affecting the soil structure, permeability, and aeration which indirectly affect the plants growth. Concentrations of both TDS and EC are generally correlated with human activities in the catchment areas. TDS varied as 65–80 and 91–238 mg/L, while EC ranged as 131–159 and 180–477 *μ*S/cm in summer and winter, successively. TDS and EC levels in both seasons were higher than reported levels from Pandoh Lake, India [32], and lower than the permissible limits set by PakEPA [30], USEPA [29], and WHO [27]. It indicated that the water from Mangle Lake was fresh having low salinity and minerals [36].

Presence of chloride ion (Cl^−^) in surface water is mainly due to atmospheric deposition, weathering of sedimentary rocks, sewage effluents, agricultural, and road run offs. It is an indicator of possible fecal contamination and a measure of the extent of dispersion of sewage discharge in water bodies [37]. High concentrations of chloride ion can make waters unpalatable and unfit for drinking and livestock watering uses [2, 36]. Mean concentrations of Cl^−^ were found to be 11 and 8.8 mg/L in summer and winter, respectively. The measured Cl^−^ levels were lower than the levels in Manchar Lake, Pakistan [38], Watland of Wadi Gaza [39], and the water quality guidelines [27, 29, 30], but higher than those found in Pandoh Lake, India [32].

### 3.2. Dissolved Concentrations of Selected Metals in Water

The average and seasonal values for selected metals are shown in Table 3. Among the selected metals, Ca (43 and 79 mg/L in summer and winter), Mg (3.4 and 4.9 mg/L in summer and winter), K (1.3 mg/L in summer and winter), and Na (2.4 and 6.8 in summer and winter) were the dominant contributors, whereas Zn and Cd (0.03 mg/L in summer and winter), Cu (0.02 mg/L in summer and winter), Mn (0.01 and 0.02 mg/L in summer and winter), and Li (0.01 in summer and winter) were the least in both seasons. The mean metal concentrations in summer were in the order: Ca > Mg > Na > K > Pb > Co > Sr > Fe > Ni > Cr > Cd > Zn > Cu > Mn > Li, while in winter the trend was slightly different: Ca > Na > Mg > K > Pb > Sr > Co > Fe > Ni > Cr > Zn > Cd > Cu > Mn > Li. Average levels of metals such as Cd, Co, Cr, Fe, K, Ni, Pb, and Zn were measured relatively higher in summer, while Ca, Cu, Li, Mg, Mn, and Sr were recorded higher in winter. The mean and median levels of Fe, Li, Mg, Mn, Na, and Zn were almost equal in summer, while Cu, Fe, Li, Sr, and Zn showed similar average and median concentrations in winter. It demonstrated that these metals showed little variations in both seasons. However, high precipitation, snow melts, large water inputs, and increasing anthropogenic activities in summer elevated the dissolved concentrations of Cd, Co, Cr, Fe, K, Ni, Pb, and Zn in surface water.

Average concentrations of the metals were compared with water quality guidelines set by national and international authorities. The maximum concentrations of Fe and mean levels of Cd, Co, Cr, Ni, and Pb were higher than the maximum permitted concentrations established by WHO [27], USEPA [29], and PakEPA [30] (Table 3). Measured concentrations of Cd, Co, and Pb were many times higher than the recommended water guidelines. In summer, 90% samples for Cd; 94% samples for Co, 63% samples for Cr, 75% for Ni, and 95% samples for Pb exceeded the water quality guidelines, whereas 89% samples for Cd and Co, 69% for Cr, 71% for Ni, and 94% samples for Pb surpassed the water guidelines in winter. Consequently, Cd, Co, Cr, Ni, and Pb emerged as the major pollutants in water samples from Mangla Lake in both seasons. It is therefore, recommended that much greater attention should be paid to the remedial measures of the emerging pollutants.

In this study, the selected metal concentrations from the water reservoir were compared with the results of other studies (Table 5). The measured mean levels of Ca were higher than those reported by Anshumali and Ramanathan [32] and lower than those reported by Shomar et al. [39] and the levels in Wielkie and Boszkowo Lakes, W. Poland [33], while K, Na, and Mg levels were lower than the reported by Mastoi et al. [38], Shomar et al. [39] and Szymanowska et al. [33]. Among the metals, Cd, Co, Cr, Cu, Ni, and Pb levels were found to be higher than reported levels by Mastoi et al. [38], Majagi et al. [40], Duman et al. [41], and Shomar et al. [39] but lower than those reported by Szymanowska et al. [33]. Iron and Zn mean that concentrations were higher than the results reported by Mastoi et al. [38] but lower than those reported by Majagi et al. [40], Duman et al. [41], Lokeshwari and Chandrappa [42, 43], and Szymanowska et al. [33], whereas Mn average concentrations were lower than those reported by Majagi et al. [40], Duman et al. [41], and Szymanowska et al. [33].

### 3.3. Source Identification of Selected Metals in Water

The correlation study was carried out to find the plausible associations of selected metals in surface water from Mangla Lake (Table 6). In summer, strong positive associations were found between Mg-Na (*r* = 0.71), while significant correlations were noted between Mg-Mn (*r* = 0.58), Mn-Na (*r* = 0.60), and Ca-Mg (*r* = 0.50). Some negative associations were also noted between Ca-Ni, Cd-K, Co-Na, K-Cr-Li, and Ni-Sr. In winter, strong positive correlation was observed between Na-Ca (*r* = 0.77) and Na-Mg (*r* = 0.68), while other positive associations were between Ca-Mg, Sr-Cr, Sr-Mg, Zn-Cd, and Cu-Cr. Some negative correlations between Co-Ca, K-Ni, Ni-Sr, and Zn-Mg were also noted. It demonstrated that the metals showing positive mutual associations were likely to be contributed by same sources, while metals showing negative associations were found to have opposite distributions in surface water.

Furthermore, multivariate principal component analysis (PCA) was employed in order to understand the complex nature of associations among the metals (Table 7). In summer, six principal components (PCs) with eigenvalues > 1 that explained about 75% of the total variance of the dataset were obtained. Principal component 1 (PC 1), which accounted for 25% of the total variance, had elevated loadings (>0.70) for Ca, Sr, Fe, and Zn, and a moderate loading for K. PC 2, which accounted for 15% of the total variance, exhibited higher loadings for Mg, Mn, K, and Na. PC 3 (10% of total variance) revealed positive loadings for Cd and Ni, whereas PC4 exhibited higher contributions of Cu, K, and Li. PC 5 and PC 6 had higher loadings for Pb and Co-Cr, respectively. Alternatively in winter, five PCs with eigenvalues > 1 that explained about 67% of the total variance of the data were obtained. PC 1, which accounted for 21% of the total variance, had strong loadings (>0.75) for Ca, Mg, Na, and Sr and moderate loadings for Cu and Fe. PC 2, which accounted for 15% of the total variance, exhibited mutual associations for Co, Cr, and Ni, whereas PC 3 had higher loadings in the favor of Cd, Co, and Pb. PC 4 and PC 5 exhibited mutual associations of K-Li and Mn-Zn, respectively. Mean levels Ca, Cu, Fe, K, Mg, Mn, and Na never exceeded the maximum permitted levels established by WHO, USEPA, and PakEPA, whereas average concentrations of Cd, Co, Cr, Ni, and Pb were higher than the recommended water quality guidelines established by the national and international authorities (Table 3). Therefore, Cd, Co, Cr, Ni, and Pb were attributed to the anthropogenic intrusions such as atmospheric deposition, agricultural activities, untreated urban, and industrial wastes [44–51].

### 3.4. Health Risk Assessment of Selected Metals in Surface Water

The human health risk assessment methodology pertaining to aquatic ecosystems has been described elsewhere [10, 52–54]. Human beings may expose to metals through three main pathways including direct ingestion, inhalation through mouth and nose, and dermal absorption through skin exposures; ingestion and dermal absorption are common for water exposure [52–54]. The numeric expressions for risk assessment were obtained from the USEPA Risk Assessment Guidance for Superfund (RAGS) methodology [52]:

(1) $$\begin{array}{c} D_{\text{ing}}=\frac{C_{\text{water}}\times \text{IR}\times \text{EF}\times \text{ED}}{\text{BW}\times \text{AT}}, \end{array}$$

(2) $$\begin{array}{c} D_{\text{derm}}=\frac{C_{\text{water}}\times \text{SA}\times K_{p}\times \text{ET}\times \text{EF}\times \text{ED}\times \text{CF}}{\text{BW}\times \text{AT}}, \end{array}$$

where *D*_ing_ is exposure dose through ingestion of water (*μ*g/kg-day); *D*_derm_ is exposure dose through dermal absorption (*μ*g/kg-day); *C*_water_ is concentration of the estimated metals in surface water (*μ*g/L); IR is ingestion rate (L/day, 2.2 for adults and 1.8 for children); EF is exposure frequency (days/year, 350); ED is exposure duration (years, 70 for adults and 6 for children); BW is average body weight (kg, 70 for adults and 15 for children); AT is averaging time (days, 25550 for adults and 2190 for children); SA is exposed skin area (cm^2^, 18000 for adults and 6600 for children); ET is exposure time (hours/day, 0.58 for adults and 1 for children); CF is unit conversion factor (L/cm^3^, 0.001); and *K*_*p*_ is dermal permeability coefficient (cm/h), 0.001 for Cd, Cu, Fe, Li, Sr, and Mn; 0.002 for Cr; 0.004 for Co, Pb, and Ni; and 0.0006 for Zn [10, 52–55].

Potential noncarcinogenic risks for exposure to contaminants were assessed by comparison of the calculated contaminant exposures from each exposure route with the reference dose (RfD) in order to produce the hazard quotient (HQ), defined as follows [52]:

(3) $$\begin{array}{c} \text{H}{\text{Q}}_{\text{ing}/\text{derm}}=\frac{D_{\text{ing}/\text{derm}}}{\text{Rf}D_{\text{ing}/\text{derm}}}, \end{array}$$

where HQ_ing/derm_ is hazard quotient via ingestion or dermal contact (unitless) and Rf*D*_ing/derm_ is oral/dermal reference dose (*μ*g/kg-day). The Rf*D*_ing_ and Rf*D*_derm_ values were obtained from the literature elsewhere [10, 52, 54, 55].

The hazard quotient (HQ) is a numeric estimate of the systemic toxicity potential posed by a single element within a single route of exposure. To evaluate the overall potential for noncarcinogenic effects posed by more than one element, the computed HQs for each element are integrated and expressed as a hazard index (HI) [52]:

(4) $$\begin{array}{c} \text{HI}=\sum_{i=1}^{n}\text{H}{\text{Q}}_{\text{ing}/\text{derm}}, \end{array}$$

where HI_ing/derm_ is hazard index via ingestion or dermal contact (unitless). When HQ/HI exceeds unity, there may be a concern for potential human health risks caused by exposure to noncarcinogenic elements [52].

Noncarcinogenic health risk assessment summary for the selected metals in the water for adults and children via ingestion and dermal routes is given in Table 8. For adults via ingestion route, the mean HQ_ing_ levels were found in the order of Co > Pb > Cd > Cr > Ni > Li > Mn > Cu > Sr > Fe > Zn and Co > Pb > Cd > Cr > Ni > Mn > Cu > Sr > Fe > Zn > Li in summer and winter, respectively. The results demonstrated that Co, Pb, Cd, Cr, and Ni were the major contributor towards noncarcinogenic risks, whereas Fe, Zn, Li, and Sr were the least. Cadmium, Co, and Pb (HQ ≫ 1.0) might pose severe adverse health effects for the adults in both seasons. Moreover, there was more risk for adults in summer than winter via ingestion route. Alternatively, the average HQ_derm_ levels were found in the sequence of Cr > Co > Cd > Pb > Mn > Ni > Li > Cu > Sr > Fe > Zn and Cr > Cd > Co > Pb > Mn > Li > Ni > Cu > Sr > Fe > Zn in summer and winter, successively. The results revealed that Cr, Co, Cd, and Pb were the main contributors towards the adverse risks and Sr, Fe, and Zn were the minor participants to pose adverse effects for the adults. However, the mean HQ_derm_ levels were very less than unity, demonstrating that the metals might pose little or no adverse risks to the local population via dermal absorption of surface water.

Conversely noncarcinogenic health risk assessment was also calculated for the most sensitive population (children). Through ingestion route, the HQ_ing_ levels were found in order of Co > Pb > Cd > Cr > Ni > Li > Mn > Cu > Sr > Fe > Zn in both seasons. The calculated HQ_ing_ levels of Co, Pb, Cd, and Cr were higher than safety limit unity, indicating that these metals were the priority pollutants through oral ingestion of surface water for children. On the contrary through dermal contact of surface water, the HQ_derm_ values were found in the order of Cr > Co > Cd > Pb > Mn > Ni > Li > Cu > Sr > Fe > Zn and Cr > Cd > Co > Pb > Mn > Mn > Li > Ni > Cu > Sr > Fe > Zn in summer and winter, respectively. Chromium, Cd, Co, and Pb were the priority pollutants, whereas Sr, Fe, and Zn were the least priority elements for children through dermal absorption of surface water in the studied area. However, the HQ_derm_ levels were found to be lower than unity, indicating that there was little or no risk for children through dermal route.

HI_ing_ and HI_derm_ were also calculated to evaluate the overall noncarcinogenic risk posed by selected metals via ingestion and dermal contact of water as a whole. For adults, Cd, Cr, Pb, and Co were found to be the major contributors to the mean values of HI_ing_ (3.7*E* + 01 in summer and 2.6*E* + 01 in winter), suggesting that these metals deserved serious health concern via ingestion route. However, the mean value of and HQ_derm_ levels (7.8*E* − 04 in summer and 6.4*E* − 04 in winter) were found to be lower than unity, demonstrating that all the selected metals posed little or no hazard to adults through dermal contacts. For children, the mean HI_ing_ values were 1.3*E* + 02 and 9.3*E* + 01 in summer and winter, respectively. Hence, Cd, Cr, Co, and Pb were the major pollutants through ingestion route.

Overall, among the selected metals, Cd, Co, Cr, and Pb emerged as priority pollutants for both adults and children through ingestion intake of surface water. However, the extent of adverse health risks was more for children than adults in both seasons, since the largest contributors towards chronic noncarcinogenic risks were Cd, Cr, Pb, and Co in the present investigation. Therefore, special attention should be paid to manage these toxic metals in the study area.

## 4. Conclusions

The present study showed diverse variations of selected metals in surface water in summer and winter from the freshwater Mangla Lake, Pakistan. The mean levels of Cd, Co, Cr, Ni, and Pb were found to be higher than national and international acceptable levels in both seasons. Moreover, their mean levels were found to be higher in summer than winter, demonstrating more risk for adults and children in summer. Multivariate PCA indicated major anthropogenic contributions of Cd, Co, Cr, Ni, and Pb in the water reservoir. Noncarcinogenic health risk assessment was carried out to find out adverse health risks for adults and children. For adults and children, Cd, Co, Cr, and Pb emerged as priority pollutants through ingestion intake of surface water in both seasons. However, children were more susceptible to adverse health risks than adults. Alternatively, the selected metals posed little or no risks to the local residents via dermal contact with surface water. The results demonstrated that the inputs of Cd, Co, Cr, Ni, and Pb should be reduced and managed on priority basis in the area. It is also suggested that the metals pollution should be considered as a vital part for future planning and management strategies for restoration of water quality of the lake reservoir.

## Figures and Tables

**Table 1:** Optimum analytical conditions maintained on AAS for the analysis of selected metals using air-acetylene flame (Shimadzu AA-670, Japan).

Metal	Wavelength (nm)	HC lamp current (mA)	Slit width (nm)	Fuel-gas flow rate (L/min.)	1% Absorption concentration (ppm)
Ca	422.7	6.0	0.5	2.0	0.08
Cd	228.8	4.0	0.3	1.8	0.02
Co	240.7	6.0	0.2	2.2	0.20
Cr	357.9	5.0	0.5	2.6	0.09
Cu	324.8	3.0	0.5	1.8	0.09
Fe	248.3	8.0	0.2	2.0	0.10
K	766.5	5.0	0.5	1.9	0.04
Li	670.7	4.0	0.5	1.6	0.05
Mg	285.2	4.0	0.5	1.6	0.007
Mn	279.5	5.0	0.4	1.9	0.05
Na	589.0	6.0	0.5	1.6	0.02
Ni	232.0	4.0	0.15	1.7	0.10
Pb	217.0	7.0	0.3	1.8	0.20
Sr	460.7	4.0	0.5	1.6	0.10
Zn	213.9	4.0	0.5	2.0	0.02

**Table 2:** Certified versus measured concentrations (mg/L) of selected metals in standard reference material (SRM 1643d).

Metal	Certified	Measured
Ca	31.04	30.26
Cd	0.00647	0.006
Cr	0.01853	0.019
Co	0.025	0.022
Cu	0.0205	0.019
Fe	0.0912	0.092
K	2.356	2.385
Li	0.0165	0.015
Mg	7.989	7.816
Mn	0.03766	0.038
Na	22.07	21.77
Ni	0.0581	0.061
Pb	0.01815	0.019
Sr	0.2948	0.286
Zn	0.07248	0.071

**Table 3:** Descriptive statistics for selected metals and water quality parameters in water samples in comparison with national/international standards (*n* = 150).

	Summer					Winter					Water quality guidelines		
	Range	Mean	Median	SD	Skew	Range	Mean	Median	SD	Skew	WHO	USEPA	PakEPA
Ca (mg/L)	14–48	43	44	5.5	−4.8	41–169	79	71	24	2.4	100	—	200
Cd (mg/L)	<0.01–0.10	0.03	0.03	0.03	1.1	<0.01–0.08	0.03	0.02	0.03	0.81	0.003	0.005	0.001
Co (mg/L)	0.01–0.50	0.25	0.24	0.12	0.12	0.01–0.41	0.16	0.13	0.12	0.92	0.04	—	—
Cr (mg/L)	<0.01–0.21	0.08	0.07	0.05	0.65	0.01–0.19	0.07	0.07	0.04	0.93	0.05	0.1	0.05
Cu (mg/L)	<0.01–0.05	0.02	0.01	0.01	0.71	<0.01–0.06	0.02	0.02	0.02	0.67	2.0	1.3	2.0
Fe (mg/L)	0.02–0.33	0.15	0.15	0.07	0.25	<0.01–0.38	0.13	0.14	0.09	0.53	0.3	0.3	—
K (mg/L)	1.2–1.6	1.3	1.4	0.08	0.32	0.61–2.0	1.3	1.2	0.31	0.75	12	—	—
Li (mg/L)	<0.01–0.03	0.01	0.01	0.01	1.0	<0.01–0.02	0.01	0.01	<0.01	0.02	—	—	—
Mg (mg/L)	3.0–3.7	3.4	3.4	0.18	0.07	3.8–8.4	4.9	4.1	1.3	1.0	50	—	—
Mn (mg/L)	<0.01–0.05	0.01	0.01	0.01	1.4	<0.01–0.06	0.02	0.01	0.01	1.4	0.1	0.05	0.5
Na (mg/L)	2.1–2.8	2.4	2.4	0.21	0.41	3.4–35	6.8	4.2	6.5	3.5	200	—	—
Ni (mg/L)	0.01–0.42	0.13	0.11	0.09	1.3	0.01–0.29	0.11	0.10	0.07	0.36	0.07	0.7	0.02
Pb (mg/L)	0.02–1.5	0.38	0.24	0.32	1.5	<0.01–2.2	0.34	0.21	0.41	3.0	0.01	0.015	0.05
Sr (mg/L)	0.07–0.29	0.19	0.21	0.07	−0.43	0.11–0.39	0.22	0.22	0.06	0.41	—	—	—
Zn (mg/L)	<0.01–0.07	0.03	0.03	0.02	0.10	<0.01–0.08	0.03	0.03	0.02	0.65	3.0	5.0	5.0
Cl^−^ (mg/L)	9.9–15	11	9.9	1.7	0.83	5.0–15	8.8	8.7	2.8	0.76	250	250	250
DO (mg/L)	3.6–4.9	4.3	4.5	0.38	−0.92	3.6–5.0	4.4	4.6	0.42	−0.29	—	—	—
EC (*μ*S/cm)	131–159	140	139	5.6	1.2	180–477	250	214	69	2.0	1500	—	—
pH	7.6–8.2	8.0	8.1	0.16	−1.2	7.4–8.3	7.8	7.9	0.33	−0.04	6.5–8.5	6.5–8.5	6.5–8.5
*T* (°C)	37–38	38	38	0.08	0.26	10–13	11	11	0.41	3.0	—	—	—
TA (mg CaCO_3_/L)	58–290	144	131	53	0.81	30–195	94	75	42	0.82	200	—	—
TDS (mg/L)	65–80	70	69	2.8	1.2	91–238	125	107	34	1.9	1000	500	1000

Reference	Present study										[27]	[29]	[30]

**Table 4:** Comparison of mean levels of water quality parameters of the present study with some other studies.

Water body	Cl^−^	DO	EC	pH	TA	TDS	Reference
	(mg/L)	(mg/L)	(*μ*S/cm)		(mg/L)	(mg/L)	
Pandoh Lake, India (monsoon)	1.83	8.75	52.7	6.26	—	28.1	[32]
Pandoh Lake, India (winter)	4.63	7.75	118	7.79	—	68.9	[32]
Pandoh Lake, India (summer)	1.53	8.08	71.6	7.35	—	53.2	[32]
Wielkie Lake, W. Poland	—	—	—	7.7	—	—	[33]
Boszkowo Lake, W. Poland	—	—	—	7.7	—	—	[33]
Domimickie Lake, W. Poland	—	—	—	7.6	—	—	[33]
Lake Beysehir, Turkey	—	9.2	350	8.0	—	—	[34]
Manchar Lake, Pakistan	431.6	—	2310	8.4	125.8	—	[38]
Watland of Wadi Gaza (summer)	924	5.3	4200	7.6	—	—	[39]
Watland of Wadi Gaza (winter)	478	8.4	2180	8.39	—	—	[39]
Mangla Lake, Pakistan (summer)	11	4.3	140	8.0	144	70	Present study
Mangla Lake, Pakistan (winter)	8.8	4.4	250	7.8	94	125	Present study

**Table 5:** Comparison of mean concentrations of selected metals of the present study with some other studies.

Water body	Ca	Cd	Co	Cr	Cu	Fe	K	Li	Mg	Mn	Na	Ni	Pb	Sr	Zn	Reference
Pandoh Lake, India (monsoon)	10.7	—	—	—	—	—	1.87	—	2.45	—	2.1	—	—	—	—	[32]
Pandoh Lake, India (winter)	24.43	—	—	—	—	—	2.46	—	6.32	—	5.51	—	—	—	—	[32]
Pandoh Lake, India (summer)	7.99						1.91	—	1.17		3.87			—	—	[32]
Wielkie Lake, W. Poland	82	7.81	13.4	5.69	5.46	110	6.9	—	15.9	4.09	—	10.7	76.5	—	—	[33]
Boszkowo Lake, W. Poland	108	11.14	24.7	7.25	5.63	180	7.2	—	15.1	2.86	—	11.2	61.2	—	—	[33]
Domimickie Lake, W. Poland	51	8.47	14.3	5.87	3.93	140	2.9	—	16.3	3.23	—	9.0	63..4	—	—	[33]
Lake Beysehir, Turkey	—	0.11	—	0.086	—	—	—	—	—	—	—	—	0.028	—	—	[34]
Manchar Lake, Pakistan	70.7	0.001	0.004		0.0089	0.012	17.6	—	56.2		521.5	0.0043	0.009	—	0.0157	[38]
Watland of Wadi Gaza (summer)	136	0.006	0.043	0.065	0.004	0.382		—	89	0.423	678		0.012	—	0.082	[39]
Watland of Wadi Gaza (winter)	102.9	0.0016	0.023	0.0206	0.0165	0.0091		—	65.7	0.267	124		0.0408	—	0.15	[39]
Karanja Reservoir, India	—	—	—	—	0.211	0.585	—	—	—	0.225	—	0.778	1.103	—	0.2103	[40]
Sapanca Lake, Turkey		0.003		0.062	0.018			—		0.023		0.046	0.036	—	0.089	[41]
Bellandur Lake, India		0.0007		0.006	0.012	1.09		—				0.003	0.009	—	0.132	[42]
Lalbagh Tank, India				0.0001	0.001	0.166		—				0.001	0.0004	—	0.043	[43]
Mangla Lake, Pakistan (summer)	43	0.03	0.25	0.08	0.02	0.15	1.3	0.01	3.4	0.01	2.4	0.13	0.38	0.19	0.03	Present study
Mangla Lake, Pakistan (winter)	79	0.03	0.16	0.07	0.02	0.13	1.3	0.01	4.9	0.02	6.8	0.11	0.34	0.22	0.03	Present study

**Table 6:** Correlation coefficients matrix (*r*) for selected metals in water samples in summer (below the diagonal) and winter (above the diagonal) from Mangla Lake (*n* = 150).

	Ca	Cd	Co	Cr	Cu	Fe	K	Li	Mg	Mn	Na	Ni	Pb	Sr	Zn
Ca	1	−0.10	−0.31	−0.17	0.15	−0.04	−0.09	0.24	0.59	0.14	0.77	0.01	−0.08	−0.03	−0.12
Cd	0.01	1	−0.11	0.01	−0.20	−0.12	0.01	0.18	−0.26	−0.16	−0.23	0.10	−0.09	−0.17	0.44
Co	−0.16	0.05	1	−0.21	−0.04	0.12	−0.12	−0.19	−0.38	−0.08	−0.31	−0.11	0.04	−0.25	0.12
Cr	0.28	0.03	−0.24	1	0.39	0.01	0.01	−0.12	0.31	−0.07	0.14	−0.14	0.30	0.58	−0.04
Cu	−0.11	0.05	0.17	−0.11	1	−0.05	−0.12	−0.06	0.25	−0.01	0.29	0.04	0.03	0.13	−0.24
Fe	0.36	0.25	−0.15	0.44	−0.01	1	0.11	0.09	0.13	−0.17	0.06	−0.17	0.08	0.28	−0.23
K	−0.14	−0.48	−0.05	−0.34	−0.14	−0.29	1	0.34	0.36	−0.15	−0.28	−0.31	0.22	0.12	0.14
Li	−0.45	−0.23	0.26	−0.51	0.35	−0.48	0.14	1	0.26	0.02	0.05	−0.10	−0.01	−0.07	0.18
Mg	0.50	−0.01	−0.31	0.32	−0.11	0.30	0.06	−0.09	1	−0.12	0.68	−0.23	0.26	0.49	−0.14
Mn	0.25	0.11	−0.12	0.21	−0.19	0.41	−0.15	−0.03	0.58	1	0.14	−0.12	−0.25	−0.15	−0.37
Na	0.13	−0.15	−0.41	0.22	−0.14	0.13	0.12	0.06	0.71	0.60	1	−0.05	0.02	0.16	−0.28
Ni	−0.45	−0.04	0.14	−0.12	0.18	−0.17	0.22	0.45	−0.09	−0.19	−0.10	1	0.04	−0.35	0.09
Pb	−0.06	−0.14	−0.11	−0.16	0.21	0.15	0.19	0.12	0.07	−0.11	0.09	0.20	1	0.20	0.15
Sr	0.28	0.43	−0.18	0.20	−0.06	0.49	−0.24	−0.32	0.09	0.11	−0.02	−0.33	0.04	1	−0.17
Zn	−0.02	0.25	0.01	−0.02	−0.27	0.42	−0.03	−0.22	0.01	0.22	−0.03	0.17	−0.07	0.32	1

*r values > 0.33 of <−0.33 are significant at *P* < 0.01.

**Table 7:** Principal component loadings for selected metals in summer and winter.

	Summer						Winter				
	PC 1	PC 2	PC 3	PC 4	PC 5	PC 6	PC 1	PC 2	PC 3	PC 4	PC 5
Eigenvalue	3.8	2.3	1.6	1.4	1.3	1.0	3.2	2.2	1.9	1.5	1.2
% total variance	25	15	10	9.2	8.6	6.7	21	15	13	10	8.0
% cumulative variance	25	40	51	60	69	75	21	36	48	59	67

Ca	0.73	0.27	0.13	0.01	0.06	−0.20	0.91	−0.17	0.01	0.004	−0.09
Cd	−0.02	−0.06	0.71	0.17	−0.29	0.05	−0.17	−0.09	0.72	0.14	0.10
Co	0.07	−0.27	0.12	0.19	−0.34	0.49	−0.27	0.37	0.44	−0.16	0.22
Cr	0.10	0.19	−0.06	0.08	−0.19	0.89	−0.02	0.90	−0.08	−0.08	0.14
Cu	0.03	−0.10	0.14	0.82	0.19	0.13	0.44	0.27	0.11	−0.38	0.02
Fe	0.69	0.25	−0.15	0.001	0.19	−0.40	0.61	0.002	−0.01	0.28	0.21
K	0.46	0.49	0.10	0.48	0.03	0.26	−0.05	0.08	0.01	0.79	0.20
Li	−0.30	0.16	0.47	0.61	0.03	0.35	0.33	−0.21	−0.25	0.61	0.03
Mg	0.01	0.85	−0.19	0.01	0.13	−0.18	0.76	0.39	0.18	0.32	0.21
Mn	0.26	0.83	−0.07	−0.09	−0.21	0.03	0.06	−0.02	0.05	−0.02	0.81
Na	−0.15	0.87	−0.01	−0.06	0.11	−0.13	0.88	0.14	0.17	−0.19	−0.08
Ni	−0.03	−0.04	0.88	0.05	0.18	0.03	0.08	0.59	−0.33	−0.27	0.23
Pb	0.06	0.02	0.10	0.22	0.85	0.04	0.03	0.26	0.65	0.04	0.11
Sr	0.70	−0.02	−0.39	−0.04	0.17	−0.08	0.77	0.08	0.23	0.24	0.10
Zn	0.68	0.06	0.28	−0.54	0.02	0.04	−0.17	−0.21	−0.37	0.15	0.52

**Table 8:** Noncarcinogenic health risk assessment summary for the selected metals in the water for adults and children via ingestion and dermal routes.

	Rf*D*_ing_ (*μ*g/kg-day)	Rf*D*_derm_ (*μ*g/kg-day)	Adults				Children			
			Summer		Winter		Summer		Winter	
			HQ_ing_	HQ_derm_	HQ_ing_	HQ_derm_	HQ_ing_	HQ_derm_	HQ_ing_	HQ_derm_
Cd	0.5	0.025	2.0*E* + 00	1.9*E* − 04	1.6*E* + 00	1.6*E* − 04	7.6*E* + 00	5.6*E* − 04	6.3*E* + 00	4.6*E* − 04
Co	0.3	0.06	2.5*E* + 01	2.4*E* − 04	1.6*E* + 01	1.5*E* − 04	9.7*E* + 01	7.1*E* − 04	6.1*E* + 01	4.5*E* − 04
Cr	3	0.075	7.8*E* − 01	3.0*E* − 04	7.5*E* − 01	2.9*E* − 04	3.0*E* + 00	8.7*E* − 04	2.9*E* + 00	8.4*E* − 04
Cu	40	8	1.4*E* − 02	3.3*E* − 07	1.7*E* − 02	4.0*E* − 07	5.3*E* − 02	9.6*E* − 07	6.4*E* − 02	1.2*E* − 06
Fe	700	140	6.4*E* − 03	1.5*E* − 07	5.8*E* − 03	1.4*E* − 07	2.4*E* − 02	4.5*E* − 07	2.2*E* − 02	4.0*E* − 07
Li	2	1	1.3*E* − 01	1.2*E* − 06	1.3*E* − 06	1.3*E* − 06	5.8*E* − 02	3.5*E* − 06	4.9*E* − 01	3.8*E* − 06
Mn	24	0.96	1.7*E* − 02	2.0*E* − 06	2.3*E* − 02	2.7*E* − 06	5.8*E* − 02	6.0*E* − 06	7.7*E* − 02	8.0*E* − 06
Ni	20	5.4	1.9*E* − 01	1.4*E* − 06	1.6*E* − 01	1.1*E* − 06	6.2*E* − 01	4.0*E* − 06	5.1*E* − 01	3.3*E* − 06
Pb	1.4	0.42	8.2*E* + 00	5.2*E* − 05	7.3*E* + 00	4.6*E* − 05	2.5*E* + 01	1.5*E* − 04	2.2*E* + 01	1.4*E* − 04
Sr	600	120	9.6*E* − 03	2.3*E* − 07	1.1*E* − 02	2.6*E* − 07	3.7*E* − 02	6.7*E* − 07	4.2*E* − 02	7.7*E* − 07
Zn	300	60	3.2*E* − 03	4.6*E* − 08	3.1*E* − 03	4.5*E* − 08	1.2*E* − 02	1.4*E* − 07	1.2*E* − 02	1.3*E* − 07

HI_ing/derm_			3.7*E* + 01	7.8*E* − 04	2.6*E* + 01	6.4*E* − 04	1.3*E* + 02	2.3*E* − 03	9.3*E* + 01	1.9*E* − 03
